# Origin, Maintenance and Variability of the Asian Tropopause Aerosol Layer (ATAL): The Roles of Monsoon Dynamics

**DOI:** 10.1038/s41598-018-22267-z

**Published:** 2018-03-02

**Authors:** William K. M. Lau, Cheng Yuan, Zhanqing Li

**Affiliations:** 1 Earth System Science Interdisciplinary Center, U. of Maryland, College Park, MD 20740 USA; 2 Department of Atmospheric and Oceanic Sciences, U. of Maryland, College Park, MD 20740 USA; 3 School of Atmospheric Sciences, Nanjing University, Nanjing, China; 4 State Key Laboratory of Earth Surface Processes and Resource Ecology and College of Global Change and Earth System Science, Beijing Normal University, Beijing, China

## Abstract

Using NASA MERRA2 daily data, we investigated the origin, maintenance and variability of the Asian Tropopause Aerosol Layer (ATAL) in relation to variations of the Asia Monsoon Anticyclone (AMA) during the summer of 2008. During May-June, abundant quantities of carbon monoxide (CO), carbonaceous aerosols (CA) and dusts are found in the mid- and upper troposphere over India and China, arising from enhanced biomass burning emissions, as well as westerly transport from the Middle East deserts. During July-August, large quantities of dusts transported from the deserts are trapped and accumulate over the southern and eastern foothills of the Tibetan Plateau. Despite strong precipitation washout, ambient CO, CA and dust are lofted by orographically forced deep convection to great elevations, 12–16 km above sea level, via two key pathways over heavily polluted regions: a) the Himalayas-Gangetic Plain, and b) the Sichuan Basin. Upon entering the upper-troposphere-lower-stratosphere, the pollutants are capped by a stable layer near the tropopause, advected and dispersed by the anticyclonic circulation of AMA, forming the ATAL resembling a planetary-scale “double-stem chimney cloud”. The development and variability of the ATAL are strongly linked to the seasonal march and intraseasonal (20–30 days and higher frequency) oscillations of the Asian monsoon.

## Introduction

The upper troposphere lower stratosphere (UTLS), situated between 200–50 hPa pressure levels (12–16 km above sea level), is the unique region encompassing the tropopause, and transition between the troposphere and the stratosphere. While the earth’s climate change is mostly measured in terms of conditions in the troposphere and at the earth surface, water vapor, ozone and other chemical constituents in the UTLS are known to have important impacts on climate at the earth surface and human health. It is well recognized that the earth’s radiation budget is sensitive to water vapor and ozone changes in the UTLS^1–8^. Recently, there have been a growing number of studies linking UTLS transport of water vapor and gaseous pollutants such as carbon monoxide to vertical motions associated with the establishment of the Asian Monsoon Anticyclone (AMA) during the boreal summer^9–15^. Measurements from lidar and high altitude balloon measurements revealed relatively high concentration of BC, and other micron and sub-micron size aerosols in the UTLS over locations in India and China during the Asian Summer Monsoon (ASM), suggesting upward transport from tropospheric sources^16–18^. Vernier *et al*. found from the Cloud-Aerosol-Lidar and Infrared Pathfinder Satellite Observations (CALIPSO), the existence of an extensive Asian Tropopause Aerosol Layer (ATAL) between 12–18 km above the earth surface spanning the Middle East to Eastern Asia [15–45°N, 0–150°E] during the ASM season^19,20^. The discovery of the ATAL has sparked a growing body of research addressing new questions regarding the origin, composition of the ATAL, transport pathways by which surface pollutants enter the ATAL, as well as possible relationships to the AMA and climate change radiative forcing.

It is well known that aerosol species from both natural and anthropogenic sources can enter the ATAL via diverse pathways, i.e., volcanic eruptions, generation of secondary aerosols through ice cloud microphysical and chemical processes, and atmospheric transport^21–23^. Our study is focused on transport processes only. Many previous studies have shown that both slow tropical upwelling and overshooting deep convection associated with the ASM can transport water vapor, as well as surface pollutants including SO_2_, black carbon (BC) and organic carbon (OC) over India and China into the UTLS^9,24–28^. More recently, Pan *et al*. using carbon monoxide as a transport tracer in global model simulations showed the importance of sub-seasonal scale convective motions in the southern flank of the Tibetan Plateau in transporting boundary layer pollutants to the UTLS during the ASM season^29^. From *in situ* measurements and model experiments, Yu *et al*. found that aerosol transport via the ATAL is a singular source which occurs during boreal summer each year, providing up to 15% of UTLS aerosols in the northern hemisphere annually^30^.

Contemporaneously, the last decade has also seen a dramatic increase in studies of aerosol-climate interactions for the ASM, revealing strong evidences that monsoon meteorology not only can affect emissions, transport, and accumulation of aerosols but can also be influenced by aerosol radiative and microphysical effects, impacting monsoon weather and climate on diverse temporal (hours to decades), and spatial (1–10^4^ km) scales^31,32^. These studies have shown that aerosol, particularly the light absorbing types, *e.g*., desert dusts, black carbon and organic carbon from biomass burning are components of an intrinsic aerosol-monsoon climate system^33,34^. While it is now well recognized that the UTLS transport of aerosols and chemical gases are closely tied to the variations of the AMA, it is not clear how the aerosol transport are related to intrinsic monsoon processes. As a pilot, this study is focused on the formation, seasonal and intraseasonal variability of the ATAL as demonstrated by the transport processes of black carbon and dust aerosols. It will be followed by investigations (ongoing) of interannual variability and multi-decadal change of the ATAL, and how these aerosols, through its light absorbing properties, may provide feedback to the ATAL transport processes. This work differs from most previous UTLS transport studies in that it is not focused on the overall composition of gases or aerosol species in the ATAL, but rather on the roles of monsoon dynamical processes, *i.e*., precipitation, monsoonal ascent, convective lofting, wet and dry removal processes, in establishing the ATAL.

Our study is based on data from the NASA Modern-Era Retrospective analysis for Research and Applications Version-2 (MERRA2), which includes assimilation of aerosol optical thickness from MODerate resolution Imaging Spectro-radiometer (MODIS) and Multi-angle Imaging Spectro-Radiometer (MISR) satellite observations, providing, 4-dimensional high-resolution (3 hourly, 0.5 × 0.625 latitude-longitude, 72 vertical levels) global data of select chemical gases (ozone, CO, N_2_O and others), aerosol species (sulfate, BC, OC, dust and sea salt) and meteorology from surface to the stratospheric, under all-weather conditions. It is important to note that the MERRA2 aerosol data represent model outputs constrained by both meteorological, and aerosol AOD observations. The meteorology and aerosol fields are dynamically consistent to the extent that the physics (radiative, latent heating and others), energy and water conservation principles governing them are largely maintained in the final assimilated fields. For the purpose of identifying monsoon dynamics, we focus on CO, carbonaceous aerosols CA BC + OC) and dust to compare and contrast their different transport properties, *i.e*., emission sources, horizontal wind advection, convective lofting, dry and wet deposition.

We focus on CO, CA (BC + OC) and dust to compare and contrast their different properties with regard to emission sources, convective lofting and washout. To ensure consistency with independent observations, MERRA2 data for rainfall, CO, aerosol vertical distributions are compared with observational estimates from Global Precipitation Climatology Project (GPCP), Microwave Limb Sounder (MLS) and Cloud Aerosol Lidar Pathfinder Satellite Observations (CALIPSO), respectively. See Methods for details of data and methodology.

## Results

**Figure 1:**
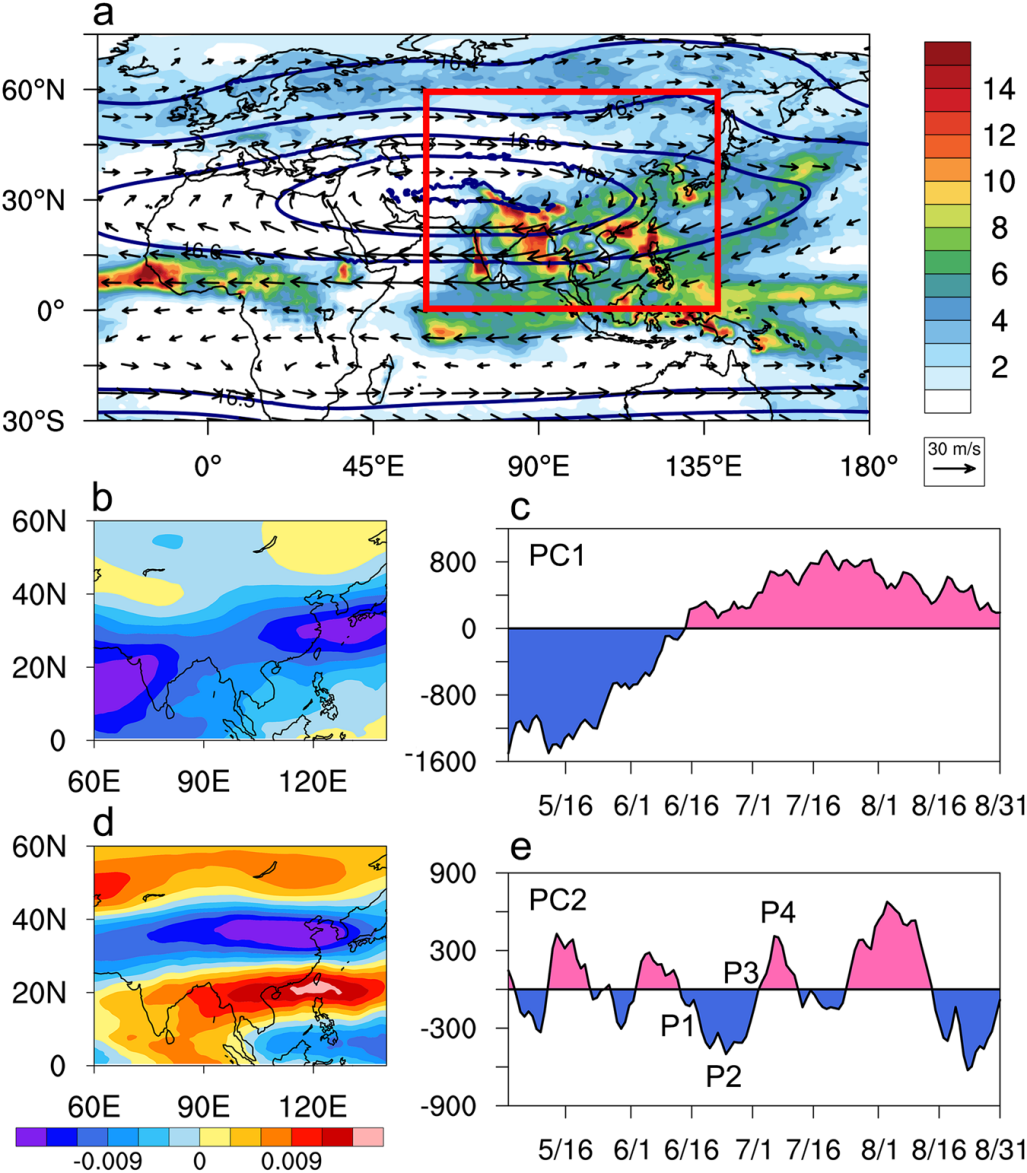
Climatological features and dominant seasonal and intraseasonal variations associated with the AMA, showing (**a**) spatial distribution of geopotential height (km) and winds (ms^−1^) at 100 hPa and rainfall (mm day^−1^) during June-July-August, 2008. Reference wind vector (30 ms^−1^) is shown in bottom right corner of panel (a). Color shading is for precipitation in mm day^−1^. Red rectangle indicates domain where EOF analysis of 100 hPa daily zonal winds was carried out. Dominant spatio-temporal patterns are shown in (**b**) EOF1, (**c**) PC1, (**d**) EOF2 and (**e**) PC2. Units of EOF and PC are scaled so that their products represent the magnitude of wind speed in ms^−1^. Maps are generated using the NCAR Command Language (Version 6.4.0, https://www.ncl.ucar.edu, 2017).

In this work, as a pilot study, we have selected the period May–August 2008 to provide in-depth analyses to unravel the relationships among sources, sink and transport of tropospheric pollutants, UTLS/ATAL processes and monsoon dynamics. The summer of 2008 is known to be a season of strong build-up of aerosols, mostly dusts over the Indian subcontinent and Arabian Sea, with heavy rain over the Himalayas foothills and northern India during June-July, followed by drought conditions over central India in July-August^34–37^. Figure 1a shows the establishment of the AMA during June-July-August (JJA) 2008, featuring a pronounced anticyclonic high pressure warm center in the upper troposphere, with strong easterlies in the deep tropics and westerlies in the extratropics spanning East Asia, the Middle East and northeastern Africa, in conjunction with heavy precipitation over the west coast of India, the Bay of Bengal, Northeastern India, and East Asia and the Philippines (Fig. 1a). These are well-known climatological features associated with the Asian summer monsoon^38–43^.

To quantify the seasonal migration and variability of the AMA, empirical orthogonal function (EOF) decomposition was first carried out based on daily 100 hPa zonal winds over the domain (60–140°E, 0–60°N, see red rectangular box in Fig. 1a). Next, we conducted composite analysis based on the first two dominant EOF modes, which explains 50.4% and 8.8% of the total variance respectively. Both modes meet the criterion for separation from sampling errors of North *et al*.^44^. The principal component of the first mode (PC1) show clearly the seasonal variations from May through August, with the 100 hPa winds in the deep tropics becoming increasingly easterlies (Fig. 1b,c) relative to the climatology. The second mode (PC2) shows quasi-periodicity of 20–30 days (Fig. 1d,e), featuring characteristic banded zonal wind structure associated with the monsoon intraseasonal oscillation (MISO)^45–49^.

### Seasonal Variation

To illustrate the seasonal variations of gases and aerosol species with respect to that of the AMA, we compare and contrast the aerosol-monsoon climatic states during the pre-monsoon (PRM) and peak monsoon (PKM) periods, defined by 10-day mean of key quantities, centered at the minimum and maximum of PC1, respectively. During PRM, heating over land surface and the lower troposphere is very strong and low-level westerlies and upper level easterlies are developing over monsoon land regions (Fig. S1a). During PKM (Fig. S1b), pronounced warming of mid- and upper troposphere and cooling in the stratosphere (relative to annual mean) is found over the Tibetan Plateau. In the following, we focus on changes on the spatio-temporal distribution of CO, CA and dust, in relationship to the AMA variations.

**Figure 2:**
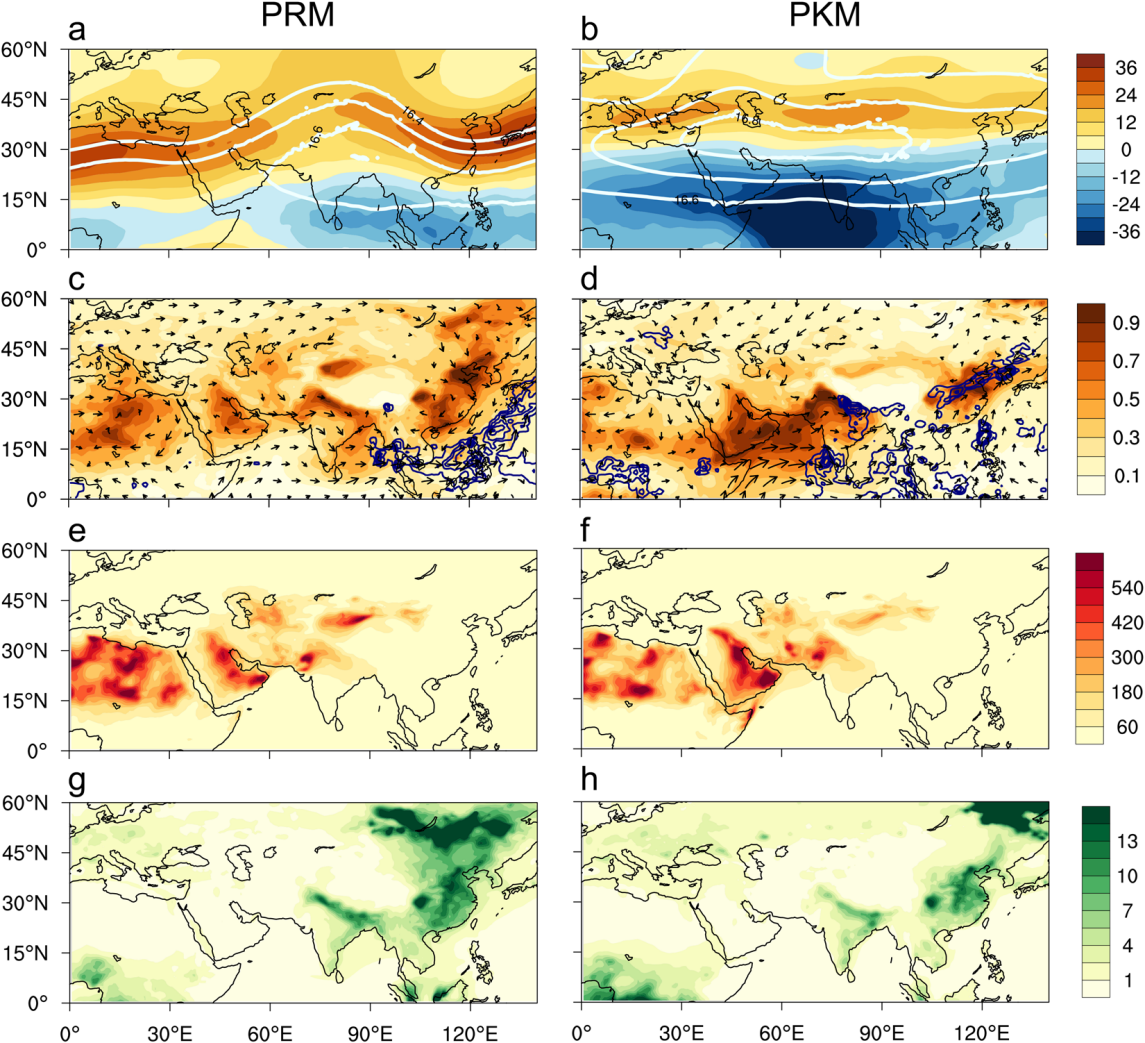
Seasonal variations of monsoon meteorology and aerosols, showing distributions of 100 hPa geopotential height (km) and zonal winds (ms^−1^) during (**a**) PRM and (**b**) PKM. Panels (c) and (d) are the same as (**a**) and (**b**) except for AOD, rainfall (contours show only rainrate >10 mm day^−1^) and 850 hPa winds. Size of reference wind vector (30 ms^−1^) is the same as shown in Fig. 1a. Panels (e) and (f) are the same as (**a**) and (**b**) except for near surface (985 hPa) dust mass concentration (ppbm). Panels (g) and (h) are for CA mass concentration. Maps are generated using the NCAR Command Language (Version 6.4.0, https://www.ncl.ucar.edu, 2017).

During PRM, the AMA at 100 hPa features a developing upper tropospheric high over South-Southeast Asia, with weak tropical easterlies in the deep tropics and meandering westerlies in the subtropics from North Africa to the East China Sea (Fig. 2a). During PKM, a strong and expansive South Asian High (SAH) anchored by the Tibetan/Iranian Plateau is established, spanning the North Africa/Middle East to the western Pacific, with pronounced easterlies in the deep tropics, and weakened westerlies in subtropics and mid-latitudes (Fig. 2b). During PRM, the monsoon land regions are mostly dry (Fig. 2c), with heavy rain and deep convections confined over the western Pacific and southern Indochina^50^. High AOD are found over a vast stretch of dust source region spanning North Africa, Arabian Peninsula, Afghanistan, the Himalayan-Gangetic Plain (HGP) of northern India, and central and northeastern China East Asian monsoon region. Both the AOD and rainfall distribution compared well with those from MODIS and GPCP (Fig. S2a,b). For AOD, the comparison is only for reassurance that the assimilation was done properly in MERRA2. For quantitative comparison, the total and fractional loading of CA and dust aerosols have been computed for the South Asian Summer Monsoon (SASM) domain (60–90°E, 10–30°N), and the East Asian Summer Monsoon (EASM) domain (100–130°E, 15–45°N) respectively. As shown in Table 1, total aerosol loading is contributed largely by dust aerosols (>80% for SASM, and >48% for the EASM). The high dust loading in the PRM atmosphere is due to enhanced emission associated with increased surface winds, deepened boundary layer, and intense dry convective mixing over the strongly heated desert surface and the Tibetan Plateau and transport by the emerging monsoon westerlies^51–54^. During PKM (Fig. 2d), heavy rain has migrated to the Himalayas-Gangetic Plain (HGP) of northern India, and to central and eastern China in conjunction of the establishment of the *Mei-Yu* rainbelt – a controlling feature of the EASM^38,55,56^. In spite of washout by heavy monsoon rainfall, total AOD increases markedly from 0.39 to 0.55 over SASM (Table 1). This is because heavy monsoon rain and wet deposition that occur during PKM tend to be intermittent and concentrated in small regions, while dust transport continues to increase due to enhanced low-level westerlies over much larger regions, as the monsoon advances^34^. For EASM, the AOD is moderately reduced from PRM (0.48) to PKM (0.34) due to precipitation washout, and weakened westerly transport from the Taklamakan Desert. The seasonal change in AOD is consistent with ground-based and satellite observations^57–60^.

**Table 1:** Domain averaged AOD and mean mass loading of carbonaceous aerosol and dust in part per billion by mass (ppbm), during pre-monsoon (PRM) and peak-monsoon (PKM) for the SASM domain (60–90°E, 10–30°N).

AOD	PRM			PKM		
	0.39			0.55		
Mean Loading	Total	Carbon	Dust	Total	Carbon	Dust
Surf- 700 hPa	89.5	1.61 (1.8%)	72.7 (81.2%)	121	0.772 (0.6%)	95.4 (78.8%)
200–100 hPa	1.58	0.24 (15.3%)	1.05 (66.4%)	3.69	0.357 (9.7%)	2.69 (72.9%)

Percentage shows fractional contribution to total aerosol loading.

Based on the near surface mass concentration, during PRM (Fig. 2e), strong dust emissions sources are found over vast desert regions west and northwest of the Asian monsoon region, and contribute mostly to the AOD signals over these regions. During PKM (Fig. 2f), dust emissions over the Middle East and Thar deserts are stronger due to the increased low-level monsoon westeries, while emissions over the northern flank of the Tibetan Plateau are weakened, due to reduced subtropical westerlies. For CA, the emission sources are very different from dust. During PRM (Fig. 2g), relative high mass loading of near surface CA, are found over Northeast and East Asia, Southeast Asia, and the HGP, near major sources of biomass mass burning, as well as industrial emissions, over East and Northeast Asia, Southeast Asia and northern India (Fig. 2g). Compared to dust, the mass fraction of CA to total aerosol are small (<10%), with higher contributions over EASM than SASM. During PKM, near surface loading of CA over Asian monsoon regions are strongly reduced due to precipitation washout, and emission quenching, except near the far northeastern domain outside the Asian monsoon rainy regions (Fig. 2h), possibly related to increased atmospheric drying, and widespread boreal regional wildfires in northeastern China and southeastern Siberia^61–63^.

**Figure 3:**
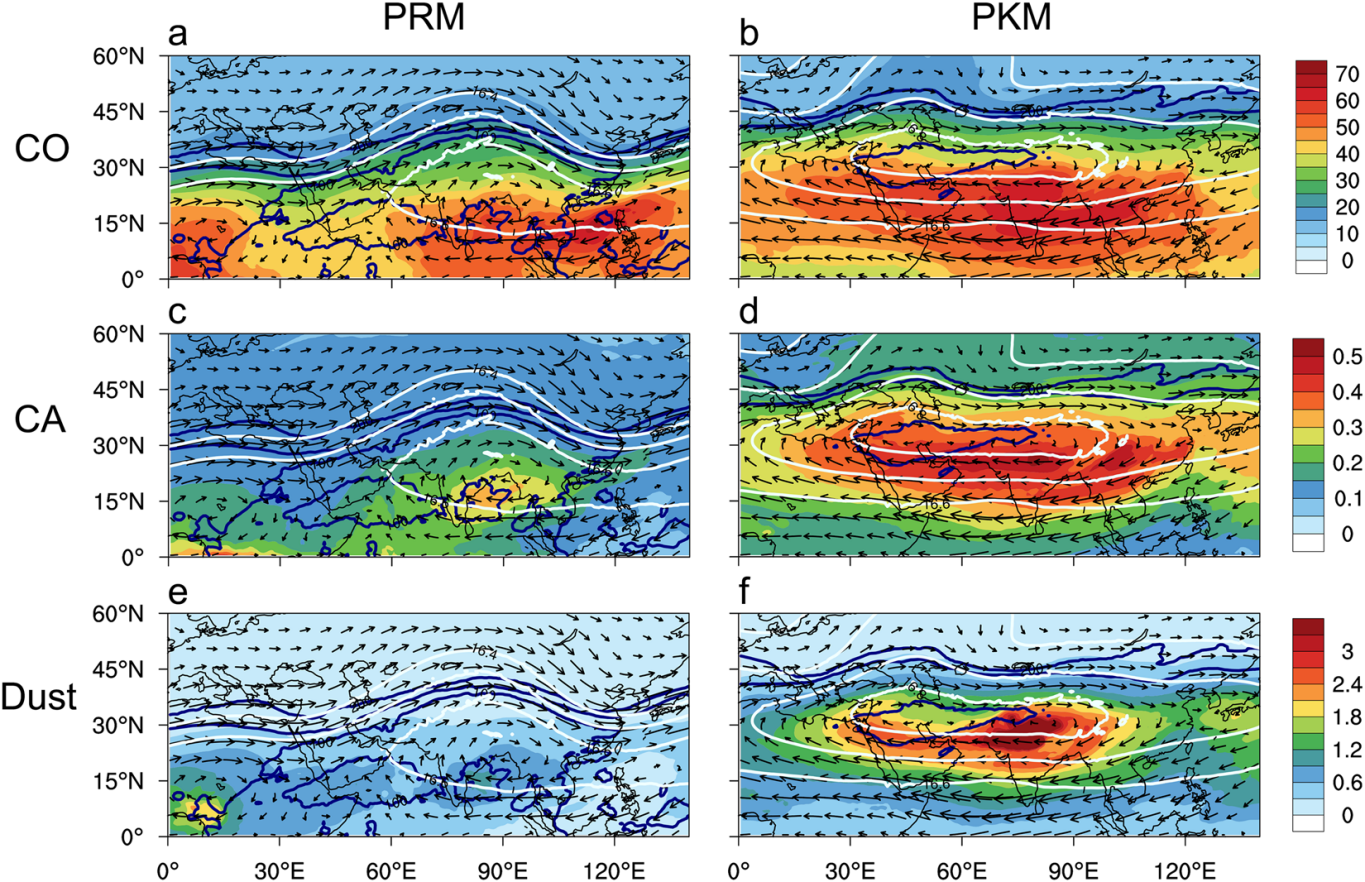
Spatial patterns of chemical gases and aerosols in relation to development of the AMA, showing distribution of CO (ppbv) at 100 hPa, superimposed with geopotential height and winds at the same level during (**a**) PRM and (**b**) PKM. Panels (c) and (d) are the same as in (**a**) and (**b**) respectively except for CA (ppbm). Panels (e) and (f) are the same as in (**a**) and (**b**) respectively, except for dust aerosols (ppbm). Maps are generated using the NCAR Command Language (Version 6.4.0, https://www.ncl.ucar.edu, 2017).

At 100 hPa, the loading patterns of CO, CA and dust are quite different from those near the surface. During PRM, high CO loading (>40 ppbv) is found spanning vast regions of Southeast Asia, and North Africa on the southern flank of the developing AMA (Fig. 3a), indicative of the well-mixed and diffusive nature of CO. In contrast, higher CA loading (>0.3 ppbm) is confined near the center of the developing AMA over the Bay of Bengal (Fig. 3c). During PKM, CO loading is substantially enhanced over the tropics 0–30°N, spanning the southern flank of the AMA (Fig. 3b). For CA, most noteworthy is the expansion of relatively confined local maximum over the Bay of Bengal during PRM, to maximum concentration (>0.5 ppbm) over vast regions of the AMA during PKM (Fig. 3d). Even more remarkable is the more than 5-fold increase in dust aerosols in the UTLS region confined by the AMA, from PRM to PKM (Fig. 3e,f). At the UTLS (200–100 hPa) dusts are mostly composed of the finest size (0.1–3 μm) particles, and contribute to a large mass fraction of aerosols in the UTLS, i.e., 66% (78%) over SASM (EASM) during PRM and 73% (64%) during PKM (See Tables 1 and 2). The MERRA2 CO patterns are in general agreement with MLS observations (Fig. S2c,d), but the magnitude is underestimated by about 10–20 percent in the deep tropics, and over 50% in the extratropics (Fig. S3a,b). Similar bias in MERRA2 CO data have been noted, and may be due to uncertainties in the estimation of biomass burning emission rates used in the MERRA2 assimilation^64,65^. There are no global CA or dust data available for comparison.

**Table 2:** Same as in Table 1, except for the EASM domain (100–130°E, 15–45°N).

AOD	PRM			PKM		
	0.48			0.34		
Mean Loading	Total	Carbon	Dust	Total	Carbon	Dust
Surf- 700 hPa	35.9	3.51 (9.8%)	17.5 (48.8%)	18.3	1.4 (7.6%)	7.7 (42.1%)
200–100 hPa	2.39	0.219 (9.2%)	1.87 (77.8%)	2.64	0.324 (12.2%)	1.7 (64.0%)

### Key transport pathways

**Figure 4:**
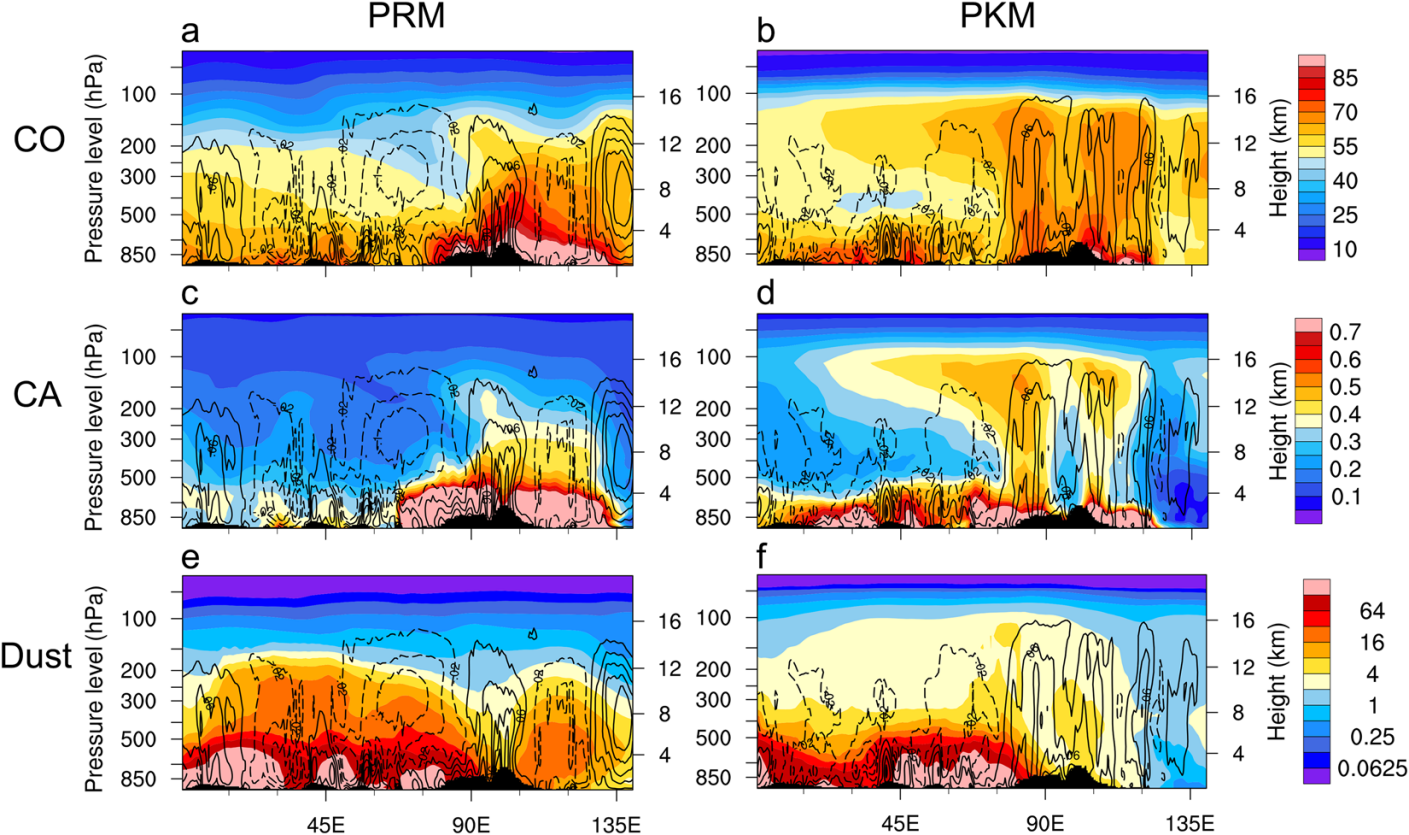
Longitude-height section (0–140°E) of CO (ppbv), averaged over the southern portion of the AMA (22–30°N) and associated vertical motion (Pa s^−1^) field with solid (dashed) contours indicating ascent (descent) superimposed, for (**a**) PRM and (**b**) PKM. Panels (c) and (d) are the same as in (**a**) and (**b**) respectively, except for CA (ppbm). Panels (e) and (f) are the same as in (**a**) and (**b**) except for dust aerosols (ppbm). Topography information is provided by ETOPO1 Global Relief Model website (https://www.ngdc.noaa.gov/mgg/global/) of National Center for Environmental Information, National Oceanic and Atmospheric Administration.

Figure 4 shows east-west cross-sections of CO, CA, dust, and vertical motions along 22.5–30°N, from surface to 50 hPa, covering the entire longitude span (0–140°E) of the AMA. In UTLS (200–50 hPa) of this region, monsoon easterlies prevail. During PRM, the accumulation and build-up of CO (Fig. 4a) is strongest near the surface, but extends throughout the troposphere up to 12 km over East Asia (90–140°E), where strong aerosol emissions from wildfires and forest clearing and burning of agricultural waste are common before the rainy season. These aerosols are transported to the UTLS by the increasing ascending motion over the monsoon region (80–130°E), and by strong dry convection from a deepened planetary boundary layer over the heated desert region (west of 80°E)^51^. During PKM (Fig. 4b), strong large-scale ascent develops over the Asian monsoon regions, and descent over the desert regions to the west. The distribution shows the shape of a “double stem chimney cloud” (DSCC), where gaseous CO is transported upward and enters the UTLS through two “stems” which collocate with the most densely populated industrial mega-complex of a) the HGP of India, and the b) Sichuan Basin (SB) of southwestern China, respectively. Strong vertical motion (Fig. 4b), and heavy precipitation (see Fig. 2d) are found over these stem regions during PKM. In the UTLS, the westward spreading and thinning of the CO layer reflects advection and diffusive mixing of CO by strong UTLS monsoon easterlies, consistent with backscatter signals detected from CALIPSO observations in the UTLS regions (Fig. S2e,f), and in agreement with the previous portrayal of the ATAL^19,20^.

Given similar emission sources to CO, the distributions of CA during PRM and PKM are similar to CO. The main difference is that CA, being fine particulate matters (~2.5 μm) in the atmosphere, can be removed from the atmosphere by dry and wet depositions, while CO as a gas is not affected by these processes During PRM (Fig. 4c), there is a strong buildup of CA in the lower-to-mid troposphere from local emissions, and dry convection in land regions of SASM and EASM. During PKM (Fig. 4d), the concentration of CA near the surface, and in the lower troposphere is significantly reduced over the Asian monsoon regions, due to precipitation wash-out. However, ambient CA particles already in the mid- and upper troposphere and those emitted during monsoon breaks, can be transported efficiently by strong ascent in the “stems” of the DSCC to the tropopause where they are capped by the stable layer across the tropopause transition region, and advected westward forming the ATAL. The vertical transport of CA appears to be stronger from the HPG stem than from the SB stem.

During PRM (Fig. 4e), dust loading is highest near the surface, and reach to high elevations up to 200 hPa, mostly likely due to increased turbulent mixing-in a deepening planetary boundary layer, and intense dry convection over the desert regions^53,66^, as well as remote transport from the North Africa desert by the strong upper tropospheric westerlies to the monsoon regions^67,68^. During PKM (Fig. 4f), dust loading in the mid-troposphere is substantially reduced, due to weakening of the upper level westerlies transport from African deserts, and removal by precipitation wash out in the monsoon regions. However, in the longitude sector (45–75°E) dust concentration remain high near the surface and in mid-tropospheric, due to the strengthening monsoon low-level south westerlies which transport dust aerosols from the Thar desert in northwestern India, and from the Middle East deserts across the northern Arabia Sea to the Indian subcontinent^33,34,60^. During PKM, while the total columnar dust loading is reduced compared to PRM due to monsoon rain washout, dust concentration is increased above 150 hPa over broad regions of UTLS. Dust transport to the ATAL is still stronger in the stems relative to other regions, but the transport appear less confined to stem regions, due to in part to the longer residence time and diffusive upwelling of ambient fine dust aerosols already present in the upper troposphere (Fig. 4e).

**Figure 5:**
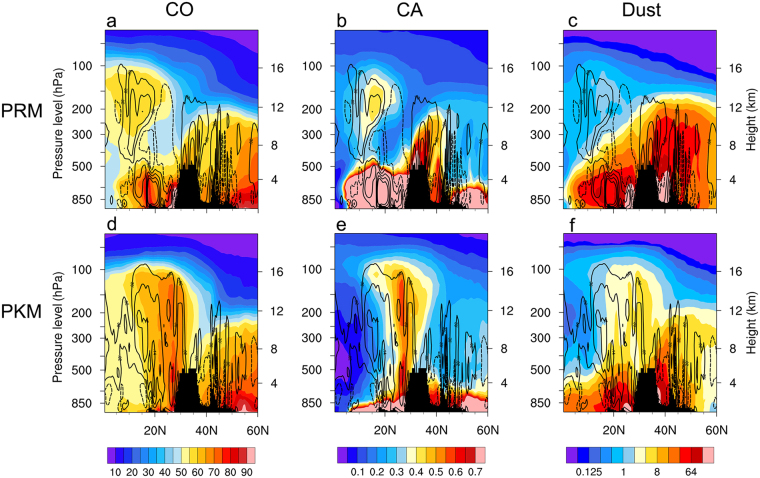
Latitude-height section (0–60°N) over the Himalayas-Gangetic Plain (82.5°E) showing the distribution of (**a**) CO, (**b**) CA and (**c**) dusts during PRM and associated vertical motion (Pa s^−1^) field superimposed, with solid (dashed) contours indicating ascent (decent). Panels (d), (e) and (f) are same as in (**a**), (**b**) and (**c**), except during PKM. Topography information is provided by ETOPO1 Global Relief Model website (https://www.ngdc.noaa.gov/mgg/global/) of National Center for Environmental Information, National Oceanic and Atmospheric Administration.

To further explore the three-dimensional structure of the DSCC, height-meridional cross-section of atmospheric constituents across the two “stem” regions, [80–85°E, 0–60°N] and [105–110°E, 0–60°N] are shown respectively for the South Asian summer monsoon (SASM) and East Asian summer monsoon (EASM). During PRM, high concentration of CO is found in the HGP “stem” and vicinity from surface to the upper troposphere (Fig. 5a), indicative of the strong vertical transport and the diffusive nature of atmospheric gases. In the deep tropics, equatorward of the TP, high CO amount (>55 ppbv) is found up to the UTLS due to remote transport and developing moist convection over Southeast Asia, during the early stage of the Asian monsoon. Likewise, heavy loading of CA is found from the surface to the lower troposphere in the deep tropics, and over the top of the TP, where strong vertical motion has developed (Fig. 5b). CA can also be detected in the UTLS in association with upward transport and easterly (westward) advection from biomass burning sources in Southeast Asia (See Fig. 3c). At this time, dust concentration is pronounced over the northern slopes of the TP, reaching up to the upper troposphere (~12 km) over Tibetan Plateau, and Taklamakan Desert and regions further north, where the prevailing westerlies, and uplifting motion by the Tibetan Plateau are still strong during the pre-monsoon season^10,52,54^. Dust concentration is also high over the Himalayas foothills, the Indian subcontinent, and reaches up to 10–12 km over regions north of the Tibetan Plateau due to westerly transport from deserts in Middle East and West Asia (Fig. 5c).

During PKM, increased CO is found from surface to the UTLS (up to ~16 km), in conjunction with strong ascent over the HGP, abutting the southern slope of the TP (Fig. 5d). For CA (Fig. 5e), the most prominent feature is a singular “chimney” with high CA concentration, reaching the UTLS, and capped near 100–90 hPa. Interestingly, this maximum injection of CA into the UTLS, occurs at the time of maximum wet scavenging and reduced biomass burning by strong monsoon precipitation as evidenced by the strong reduction (compared to PRM, Fig. 5b) of CA from surface to the lower troposphere. Apparently, ambient CA already present in the mid- and upper troposphere, above the region of maximum wet scavenging, are efficiently transported to the UTLS by strong deep convection ascent during PKM. See following subsection on wet removal vs. convective lofting of aerosols entering the ATAL, for further discussion of this topic. For dust, the overall concentration near the surface and in the troposphere is substantially reduced during PKM (Fig. 5f), compared to PRM (Fig. 5c), due to rain washout. However, as discussed previously, increased low level southwesterly over India, and deep westerlies over west China north of the Tibetan Plateau continue transport dusts from the deserts, keeping the dust amount relatively high over the region during PKM. Similar to CA, ambient fine dust already present in the mid- and upper troposphere, are transported upward into the UTLS, and enter the ATAL via the HGP “stem”. Deep convection at the top of the Tibetan Plateau may also play a role in uplifting local dust reaching the ATAL^52,69^.

**Figure 6:**
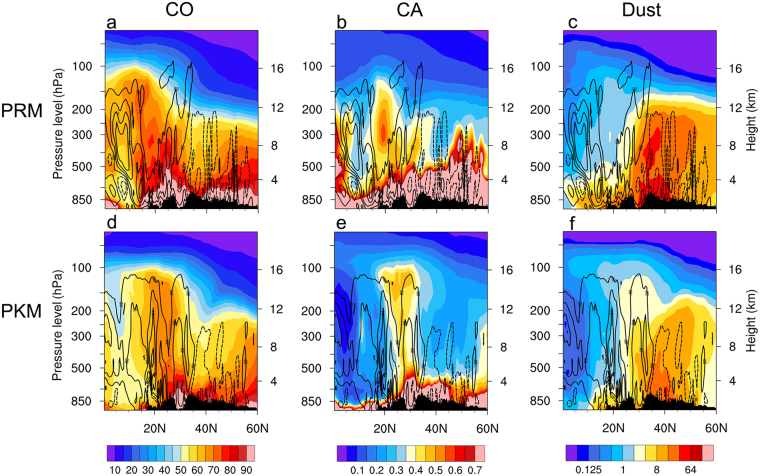
Latitude-height section (0–60°N), over the Sichuan Basin, southwestern China (105°E), showing distributions of (**a**) CO, (**b**) CA and (**c**) dusts during PRM, superimposed with vertical motion (Pa s^−1^) with solid (dashed) contours indicating ascent (decent). Panels (d), (e) and (f) are same as in (**a**), (**b**) and (**c**), except during PKM. Topography information is provided by ETOPO1 Global Relief Model website (https://www.ngdc.noaa.gov/mgg/global/) of National Center for Environmental Information, National Oceanic and Atmospheric Administration.

For the SB “stem”, the overall transport pattern is similar to that for HGP. The following discussion is focused on the differences between the two regions. During PRM (Fig. 6a), the region of high CO mass loading is more expansive than that for HGP, filling the troposphere up to the UTLS in the deep tropics (12–16 km), and midlatitudes (8–10 km), as a result of industrial emissions over the SB and regions further north, as well as remote transport from biomass burning sources over Southeast Asia. Similarly, CA is abundant from surface to mid-troposphere, increasing steadily poleward, reaching up to 8 km in the drier region to the north (Fig. 6b). Dust loading is high and reaches up to 10–12 km in northern latitudes (30–60°N), due to prevailing tropospheric westerlies, which increases transport from deserts to the region. During PKM, the upward transport pathway of CO and CA (Fig. 6d,e) becomes more well defined over the densely populated industrial mega-cities of the SB on the eastern foothills of the TP, where the occurrence of intense monsoon cyclones with deep convection and heavy precipitation are common during the peak EASM season^55,70^. In this region, vertical transport in the stem of high CA, from the surface reaching the UTLS is very pronounced, as are the reduction of CA at the surface and in the lower troposphere due to rain washout (Fig. 6e). Similar to SASM, the overall dust amount is substantially reduced over the EASM from PRM (Fig. 6c) to PKM (Fig. 6f), because of the weakening subtropical and mid-latitudes westerlies north of the TP, except in the “stem region” of the SB, where strong convective transport more dust into the ATAL.

### Monsoon Intraseasonal Oscillations (MISO)

**Figure 7:**
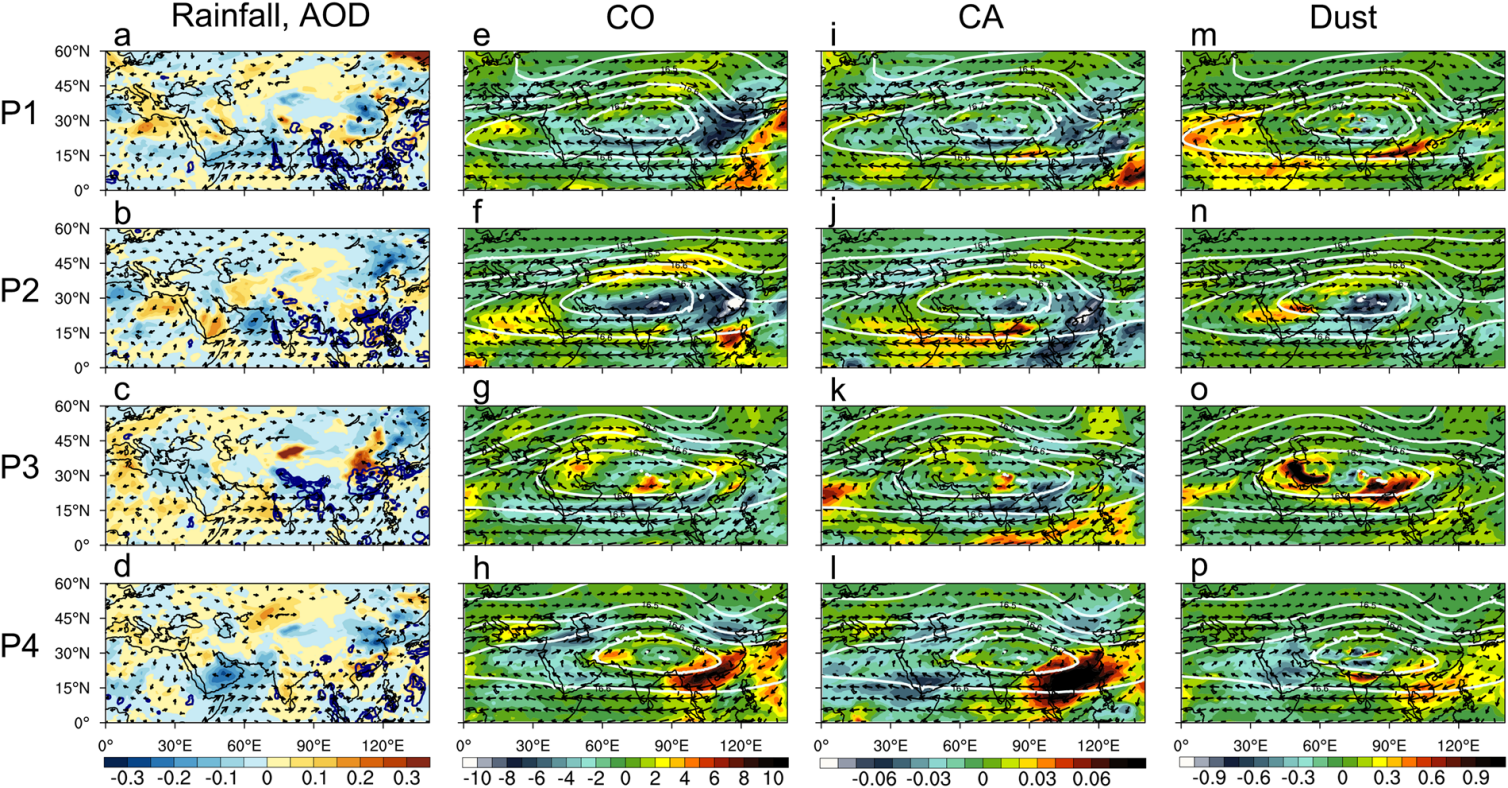
Composite distribution of total rainfall, 850 hPa winds and AOD anomalies during different phases (P1 through P4, as defined by PC2) of the Asian monsoon intraseasonal oscillations for (**a**) P1, (**b**) P2, (**c**) P3 and (**d**) P4. Only positive values are shown for rainfall. Panels (e) through (h) are the same as (**a**) through (**d**), except for CO (ppbv) at 100 hPa, with geopotential height and winds at the same level superimposed. Likewise, panels (i) through (l) and panels (m) through (p), are the same as panels (e) through (h), except for CA anomalies (ppbm) and for dust anomalies (ppbm) at 100 hPa. Maps are generated using the NCAR Command Language (Version 6.4.0, https://www.ncl.ucar.edu, 2017).

To examine the co-variability of MISO and ATAL, we have constructed composite maps of rainfall, AOD, AMA geopotential and winds, CO, CA and dust based on 4 phases of the 20–30 day oscillations, P1 through P4, delineating by the times of maximum, minimum and zero-crossings in PC2 (Fig. 1e). From Fig. 7a–d, these phases correspond to the well-known active and break cycles of the Asian monsoon^48,71–73^. The cycle starts with the development of heavy rain (>10 mm/day) over oceanic regions of the tropical western Pacific and Southeast Asia, but a lack of heavy rain on monsoon land (P1, Fig. 7a) reflecting a monsoon break situation, followed by a progression northwestward of heavy precipitation marginal monsoon land regions of Southeast Asia and western India, but over key SASM and EASM land domains, heavy precipitation is still lacking (P2, Fig. 7b). Subsequently, maximum heavy precipitation develops over the northeastern India/Himalayan foothill and Bay of Bengal, and central eastern China (P3, Fig. 7c), indicating the active phase of the monsoon. The heavy rain over these regions eventually fizzles into isolated intense rainfall cells (P4, Fig. 7d). This active/break cycle repeat itself, 4–5 times from May-August 2008 (See Fig. 1e).

At 100 hPa, the variations of CO follow closely those of the MISO of AMA circulation. During P1 (Fig. 7e), a large reduction in CO is found in the eastern portion of the AMA is associated with the equatorward advection of CO-poor air mass from the mid-latitudes (see distribution of CO in Fig. 3a,b) via a distinctive trough development in the northeastern corner of the AMA. The large increase in CO in the southeastern flank of the AMA is associated with strong upward transport of ambient CO by increased ascent associated with deep convection from heavy precipitation. These transport process continues during P2, with the negative CO anomaly advected by the AMA mean easterlies over western China and India (Fig. 7f). At the same time CO-rich air is advected around the outer flanks of the AMA, as the trough weakens. During P3 (Fig. 7g), the AMA is maximally developed, in concert with the heavy rain over the HGP, and the *Mei-yu* rainband of the Asian monsoon. However, maximum upward CO flux to the ATAL is delayed until P4 (Fig. 7h), because CO emissions from biomass burning are reduced by heavy precipitation.

For CA, the overall variations associated with the MISO (Fig. 7i–l) are similar to CO (Fig. 7e–h). The main difference is that precipitation washout strongly affects CA, but not CO. During P4, heavy rainfall and washout subside, the maximum injection of ambient CA is found in regions of strong vertical motion in the southeastern corner of the AMA. During P1 and P2 (Fig. 7m,n), dust concentration at 100 hPa is increased in the eastern, southeastern and southwestern flank of the AMA, but reduced over the main monsoon regions, indicating advection of pre-existing dust by the anticyclonic AMA circulation, as well as upward transport from sources region over the desert, west of 60E. The dust concentration is highest during the maximum active phase, P3 (Fig. 7o), showing increased loading over the southeastern corner, and the eastern portion in the inner core of the AMA. Over the monsoon region, in spite of the strong wet removal due to heavy precipitation, the strong vertical motions in the upper troposphere transport ambient dust in the mid-troposphere to the ATAL. By P4 (Fig. 7p), the dust amount at 100 hPa is strongly reduced by dry deposition and dispersed by the AMA circulation.

### Convective lofting vs. wet scavenging

**Figure 8:**
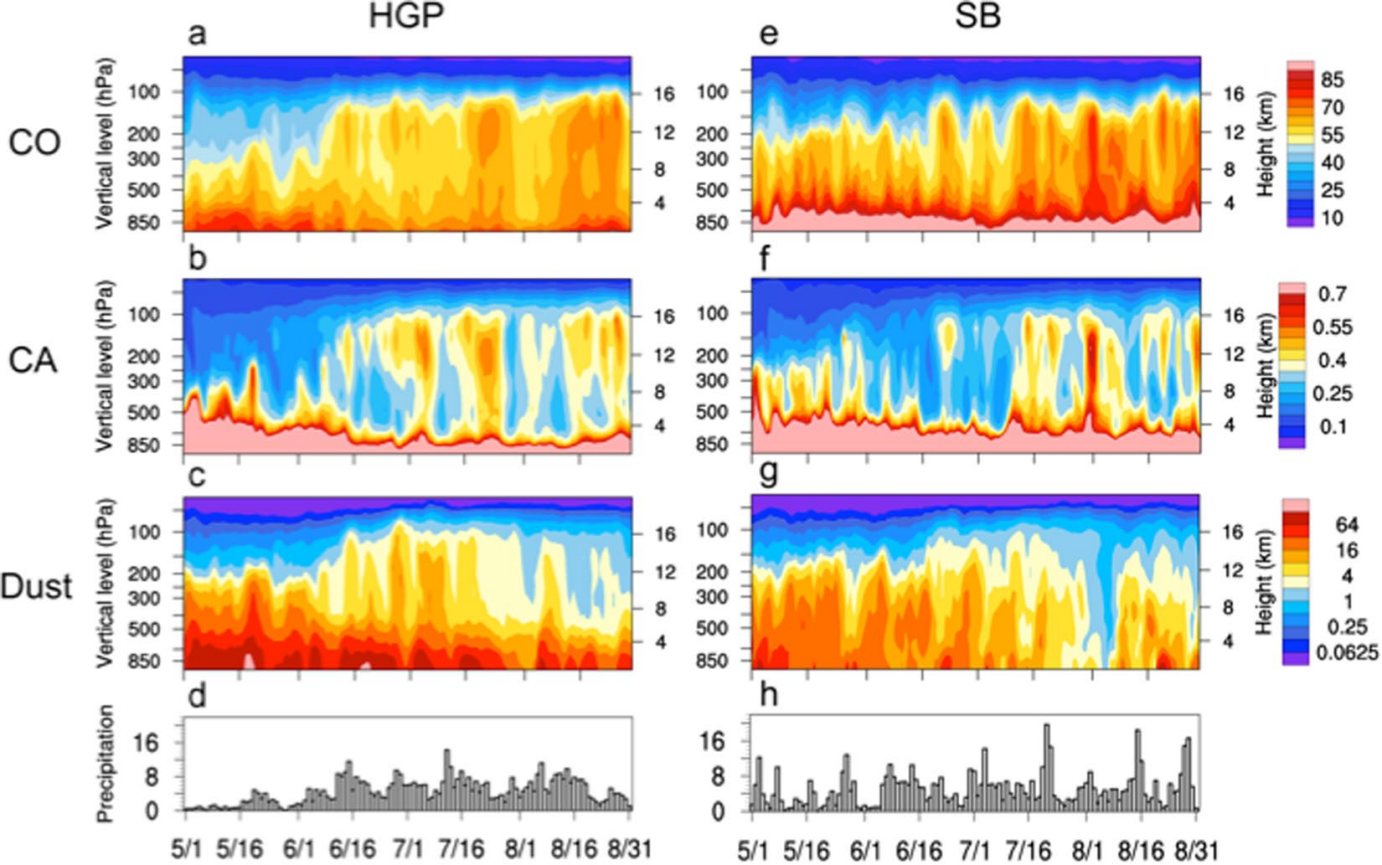
Time-height cross-section showing daily variations of (**a**) CO (ppbv), (**b**) CA (ppbm), (**c**) dust (ppbm) and time series of (**d**) precipitation (mm day^−1^), over HGP from 1 May through 31 August, 2008. Panels (e), (f), (g) and (h) are the same as in (**a**), (**b**), (**c**) and (**d**) respectively, except for SB. Plots are generated using the NCAR Command Language (Version 6.4.0, https://www.ncl.ucar.edu, 2017).

The transport of near-surface aerosols to the ATAL is dependent not only on the source emission rates and wind transport, but also on the rate of removing aerosols from the atmosphere. Because of heavy rain, wet scavenging is the major sink of ambient aerosols in ASM regions. In this subsection, we examine the competing influences of deep convection and wet scavenging in transporting aerosol species in the ATAL in the “stem regions” of the HGP and SB respectively. The HGP is a source region of CO and CA from industrial pollution, anthropogenic biomass burning, as well as from natural biogenic emissions, and wildfires. It is also a region with complex surface terrain conducive to orographic forcing of strong overshooting deep convections and heavy precipitation^34,74,75^. Here, CO and CA concentrations are relatively high from surface to 300 hPa during the pre-monsoon period (Fig. 8a,b), due to dry convection, and increased emission over the increasing hot land before the onset of heavy rainfall (June 13–14). Following the onset, CO and CA in the UTLS (200–100 hPa) increase abruptly and fluctuate approximately in phase with MISO of precipitation (Fig. 8a,b and d). Compared to CO, the lofting of CA into the UTLS appear more episodic, reflecting the competing influences of transport by overshooting convection and removal by wet scavenging. Because wet scavenging occurs in cloud (rainout) and below cloud (washout)^76–78^, aerosol removal is more efficient in the middle and lower troposphere, while upward transport by penetrative convection prevails in the UTLS region. Near the surface and in the lower troposphere below 500 hPa, both CO and CA decrease as the monsoon advances, due to quenched surface biomass burning and biogenic emissions by the increasing monsoon precipitation.

For dust, the HGP is not a source, but rather a receptor region. During the pre-monsoon period, dust aerosols transported by the monsoon southwesterlies from the deserts of the West Asia, and the Middle East piles up against the steep slopes of the Himalayas and accumulate over the HGP^34,58,79–81^. Before the onset of the Indian monsoon (June 13–14), dust concentration is high from the surface to 300 hPa (Fig. 8c), due to strong surface emission, and local dry convection, as well as long-range westerly transport from deserts of the Middle East, West Asia and as far as the North Africa^66^. After monsoon onset, dust loading in the mid- and lower troposphere decreases due to strong wet scavenging. Near the earth surface and in the lower troposphere, dust loading remains high, decreasing only slightly due to replenishment from increasing southwesterly transport from the Middle East deserts across the Arabian Sea^80,81^. During PKM, heavy precipitation is more frequent, and wet removal of accumulated dust in monsoon regions become important. However, more dusts are found in the UTLS as ambient dusts in the mid- and upper troposphere above region of strong wet scavenging are lofted into the UTLS by penetrative deep convection (Fig. 8c). Examination of atmospheric dust loading as a function of size distribution (Figs S4, S5) shows that dust particles of all sizes (0.1–10 μm) are subject to wet scavenging by precipitation, with higher rate of removal for large-size dust particles, while lofting is more efficient for smaller size dust particles. However, very fine dust particles (0.1–1 μm) represent only a small fraction of total dust content in the atmosphere over the region. As a result, dust aerosols in the UTLS consist mostly of fine dust to moderate size (radius ~1–3 μm).

Similar to HGP, SB is a region of dense population, surrounding by complex high mountain terrains in the eastern foothills of the Tibetan Plateau, with strong CO and BC emissions from both natural and industrial sources. The region is strongly affected by westerly transport and deposition of dusts from the Taklamakan desert, north of the Tibetan Plateau during the pre-monsoon season^52,82^. Here, the variability of CO, and CA are similar to HGP, indicating influences of slow seasonal rising motion, fast penetrative deep convection and wet scavenging, resulting in increasing frequency of CO and CA reaching the ATAL by deep convection from PRM to PKM. Total dust loading over SB declines from PRM to PKM (Fig. 8g) due to weakening of the tropospheric subtropical westerlies (See Fig. 2b). However, even with reduced total loading during PKM, dusts reach higher into the UTLS region (>14–15 km) by deep convective lofting. The main differences compared to HGP are that the monsoon precipitation onset over SB is less well defined, and the intraseasonal oscillations of precipitation (Fig. 8d,h) and convective lofting have higher frequency of variability.

## Discussion

Based on NASA MERRA2 reanalysis, we have carried out an in-depth analysis of the seasonal and sub-seasonal co-variability of CO and selected aerosol species (CA and dust) in the UTLS, with near surface aerosol sources and sinks, and transport during the Asian summer monsoon of 2008. During the pre-monsoon period, atmospheric loading of CO, and CA from biomass burning and desert dusts are strongly increased from surface to the mid- and upper troposphere (~5–10 km) likely due to a deepened planetary boundary layer and intensified dry convection associated with strong land surface heating, as well as orographic uplifting. During the peak monsoon season, increasing low-level westerlies transport more dusts, which are trapped by local topography and accumulated to high concentration in “aerosol receptor” regions of the Asian monsoon^34,78^. In spite of removal by increased precipitation washout, ambient aerosols in the mid- and upper troposphere are transported to the ATAL (~12–16 km) region by increased vertical motion associated with deep convective motions. Two preferred regions of strong vertical transport of CO, CA and dust include: a) the Himalayas Gangetic Plain (HGP) regions in northern and northeastern India and b) the Sichuan Basin (SB) of southwestern China, both are mega-cities complex, with dense population heavy industrial pollutions, near the southern and eastern foothill regions of the Tibetan Plateau respectively. Upon entering the UTLS, CO, CA and dust are strongly advected and dispersed by the anticyclonic flow of the AMA. These two “stem” regions, together with AMA transport pattern creates the appearance of a “Double Stem Chimney Cloud’ (DSCC, as shown in Fig. 4) portraying the spatial structure of the ATAL. Chemical gas and aerosol transport through the “stems” of the DSCC, and distributions within the ATAL fluctuate in concert with vertical and horizontal motions associated with intrinsic monsoon intra-seasonal (~20–30 days) oscillations, and higher frequency ASM variability in deep convection and precipitation. In the stem regions, while total aerosol loading of CA and dusts are reduced during peak monsoon due to wet scavenging, ambient aerosols in the mid- and upper troposphere already build up during the pre-monsoon period, continue to increase due to the large-scale rising motion associated with the monsoon seasonal advance, and get lofted into the ATAL by strong vertical motions, associated with penetrative deep convection and heavy precipitation during the peak monsoon season.

This research has shed new light on physical processes transporting pollutant from the surface to the UTLS region, in relation to the seasonal and intra-seasonal variability of the Asian aerosol-monsoon climate system^32,34^. Because of the large variance (~50%) explained by the seasonal mode, the pre-monsoon and peak-monsoon description of the ATAL is likely to be robust, occurring on annual basis. However, it needs to be confirmed by further work. More research is needed to identify and better understand the ATAL variability in connection with inter-annual, and longer-term changes of the monsoon seasonal transition and MISO. Most important, this study opens up new avenues for further investigation of fundamental atmospheric processes, including not only transport of chemical gases and aerosols, but also formation of secondary aerosols through ice-cloud nucleation, affecting UTLS aerosols, water vapor as well as aerosol-cloud radiative feedback processes, impacting climate variability and change in Asian monsoon regions, and global water and energy balance. Last but not least, it should be pointed out that MERRA2 data could be model dependent. To confirm reliability of the present results, further model and data intercomparison studies, and ultimately field observations involving high-altitude balloons and airborne measurements over the critical regions identified in this study have to be conducted.

## Methods

### MERRA2 and ancillary data

We use the NASA MERRA2 reanalysis which include aerosol data assimilation based on MODIS and MISR satellite retrievals of aerosol optical depth^83^. MERRA2 uses the latest version of the GEOS-5 global climate model and physical packages including aerosol radiation and microphysics, emissions, transport, dry and wet deposition of aerosols, as well as CO and BC emissions of biomass burning derived from MODIS estimates. It allows analyses of spatio-temporal co-variability of 4-dimensional global dynamically consistent distributions of major aerosol species, i.e., black carbon (BC), organic carbon (OC), dust, sulfate and sea salt, and meteorology from the earth surface to the UTLS, under all weather conditions and with high-resolution (3 hourly, 0.5 × 0.625 latitude-longitude, with 72 vertical levels). In this paper, we focus only on CO, carbonaceous aerosols, and light-absorbing aerosols (BC, OC and dust), to explore the relationships among ATAL variability, UTLS transport processes and monsoon dynamics. In subsequent (ongoing) work, the effects dynamical feedbacks induced by light-absorbing aerosols will be investigated. CO, a well-mixed chemical gas and a byproduct of industrial pollution and biomass burning has been widely used as a tracer for atmospheric wind in chemical transport studies^29^. The amount of CO in the atmosphere is directly related to surface emission and atmospheric wind transport and mixing, but not affected by chemical reaction, nor by dry or wet removal. On the other hand, CA and dusts are strongly affected by both dry and wet deposition, in addition to transport by winds. CO and CA have common emission sources from industrial pollution and biomass burning, while dusts are mostly from natural sources in neighboring deserts. Abundant quantities of CO, CA and dusts are found in Asian monsoon regions during the boreal spring and summer. Hence studying how monsoon rainfall and circulations affect the sources and sinks and transport of these three species (CO, CA and dusts) of surface pollutants to the UTLS can provide useful insight into the origin, maintenance and variability of the ATAL. To reassure consistency with independent observations, the MERRA2 data for rainfall, CO, aerosol vertical distributions are compared with observational estimates GPCP, MLS at 100 hPa, and CALIPSO vertical profiles of aerosol backscatter, respectively.

### Empirical Orthogonal Function (EOF) Analysis

To identify the dominant modes of seasonal and monsoon intraseasonal oscillations (MISO), the EOF analysis was carried out for 100 hPa zonal winds over the inner domain marked in Fig. 1, from May 1- August 31, 2008, using the NCAR Command Language (NCL) EOF coding package available at https://www.ncl.ucar.edu/Document/Functions/Contributed/. Composite analysis based conduced based on the principal components (PC) of the first two EOFs which are well separated from sampling noise based on the criterion of North *et al*. [1979] Composite patters for temperature, geopotential height^44^, winds, rainfall, AOD, concentrations of CO, CA and dust for the pre-monsoon (PRM) and peak monsoon (PKM) periods were constructed based on 10-day means centered on the minimum (maximum) of PC1. For MISO, we define four phases, P1 through P4, based on the 3-day mean centered at the local maximum, minimum and zero crossings of PC2 (Fig. 1e).

### Data Availability

MERRA2 reanalysis data are available at https://disc.sci.gsfc.nasa.gov/daac-bin/FTPSubset2.pl. The datasets processed and/or analysed during this study are available from the corresponding author upon reasonable request.

## Electronic supplementary material

Supplementary Information
