# Supplementary material for: Origin, Maintenance and Variability of the Asian Tropopause Aerosol Layer (ATAL): The Roles of Monsoon Dynamics
Source: Sci Rep. 2018 Mar 2;8:3960. doi: 10.1038/s41598-018-22267-z (PMC5834455; doi:10.1038/s41598-018-22267-z)
Supplement: Supplementary file 1 — Supplementary Information [file 41598_2018_22267_MOESM1_ESM.docx]

**Origin, Maintenance and Variability of the Asian Tropopause Aerosol Layer (ATAL): The roles of monsoon dynamics**

William K. M. Lau, Cheng Yuan and Zhanqing Li

**Supplementary Information**


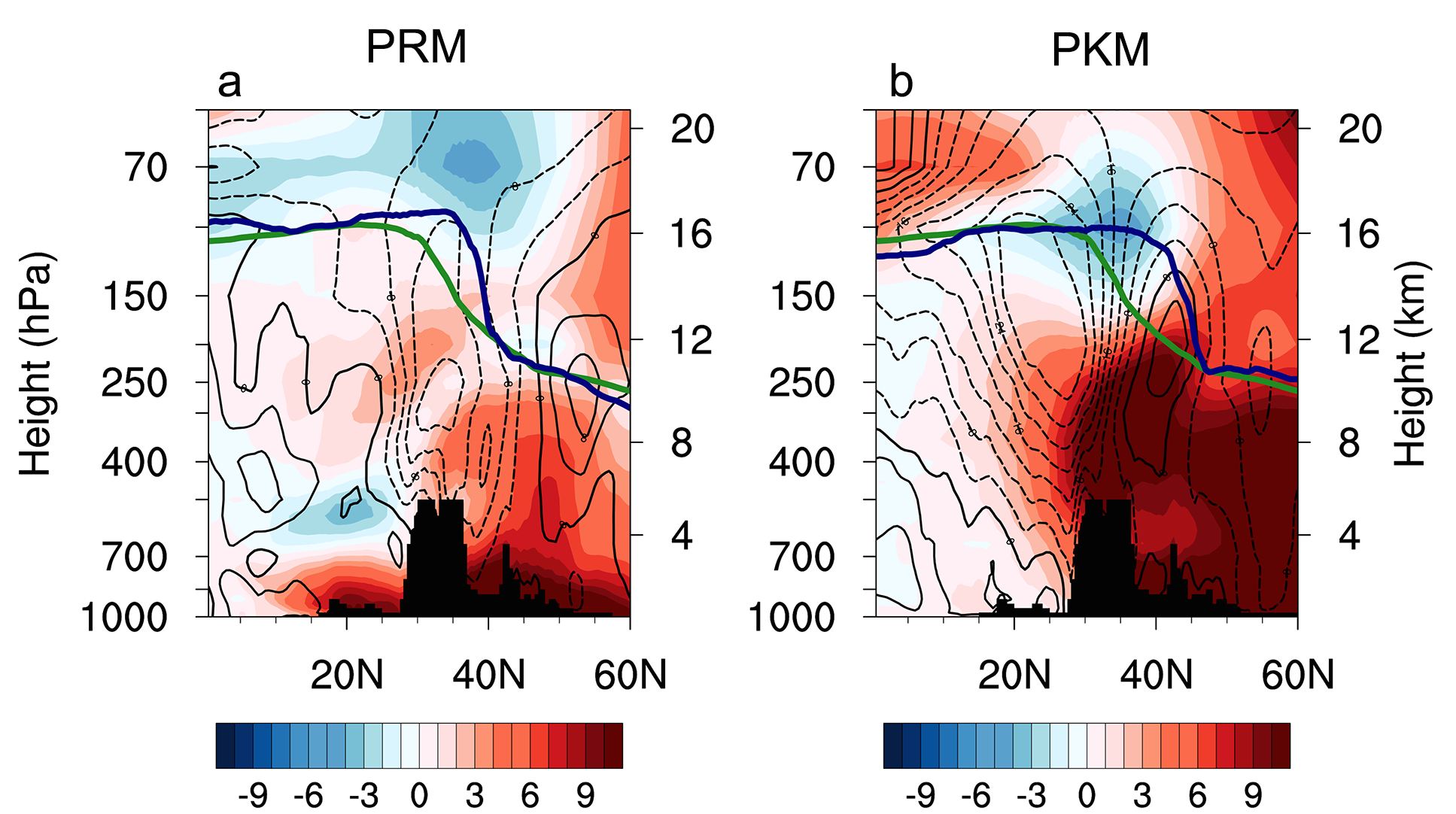


Figure S1. Latitude-height cross-section of temperature (^o^C, color shaded) and tropopause height (thick blue line) and zonal winds (ms^-1^) with positive (negative) contours indicating westerlies (easterlies), over the Indian subcontinent (82.5^o^ E) during a) PRM and b) PKM. Thick green line indicates the annual mean tropopause height. For temperature, and zonal winds, the annual means have been subtracted. Topography information is provided by ETOPO1 Global Relief Model website (<https://www.ngdc.noaa.gov/mgg/global/>) of National Center for Environmental Information, National Oceanic and Atmospheric Administration.


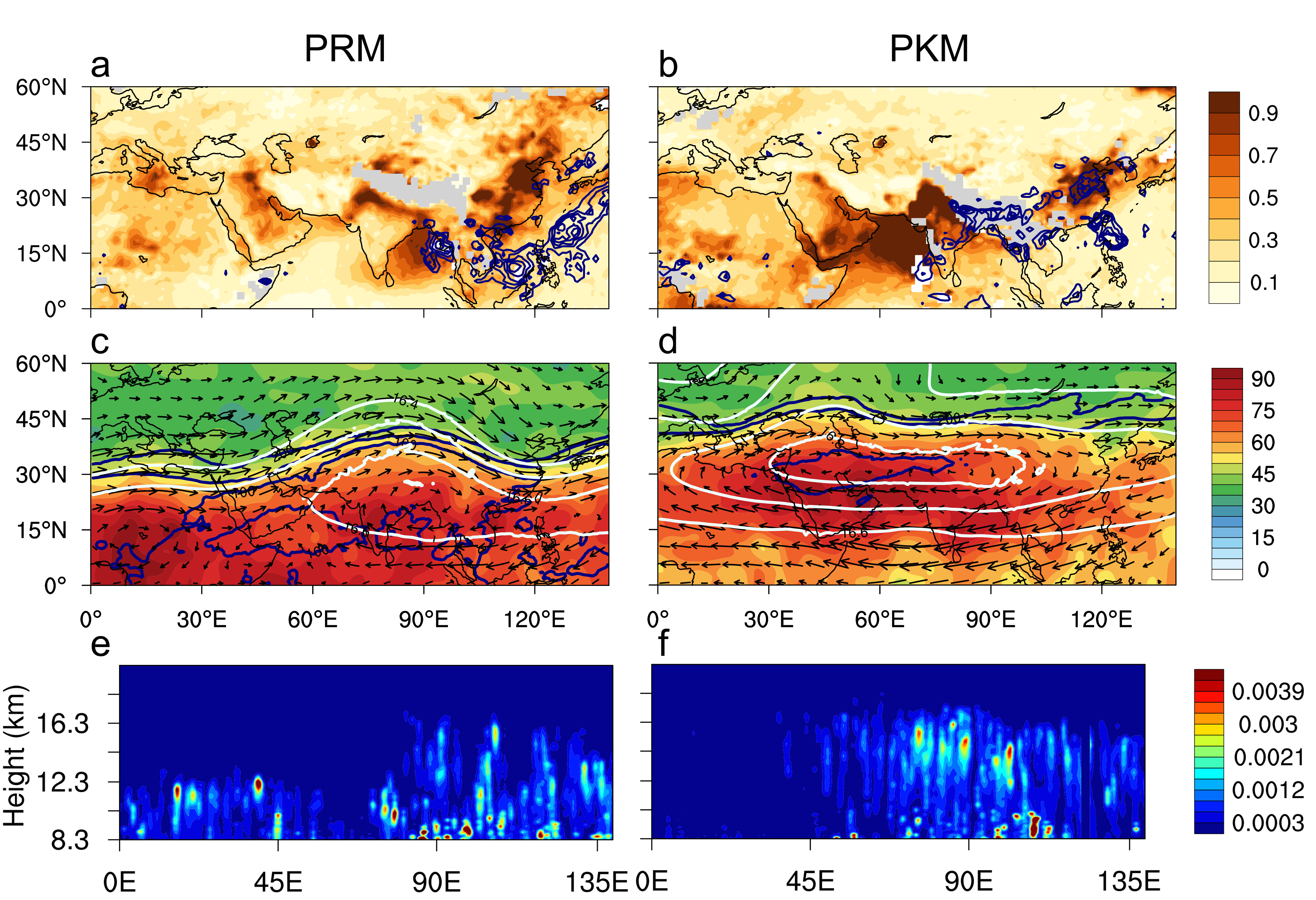


Figure S2. Spatial distribution of MODIS AOD (color) from MODIS, and TRMM rainfall (>15 mm day^-1^ contours) during a) PRM and b) PKM. Panels c) and d) are the same as in a) and b), except for CO (ppbv) from MLS, with 100 hPa geopotential height and winds superimposed. Panel e) and f) shows east-west cross-section of aerosol backscattering signals (km-1 sr-1), from CALIPSO, during PRK and PKM respectively. Maps are generated using the NCAR Command Language (Version 6.4.0, [https://www.ncl.ucar.edu](https://www.ncl.ucar.edu/), 2017).


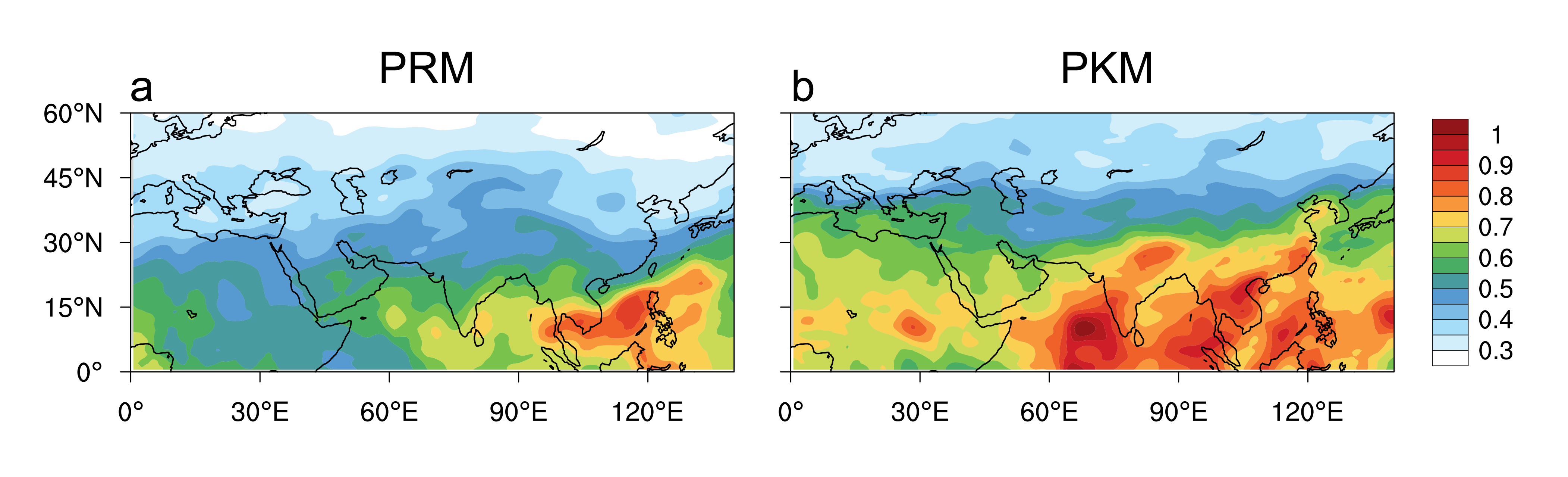


Fig. S3 Spatial distribution of the ratio of mass loading of MERRA2 CO to MLS CO, during pre- monsoon (PRM) and peak monsoon (PKM) periods, May-August, 2008.


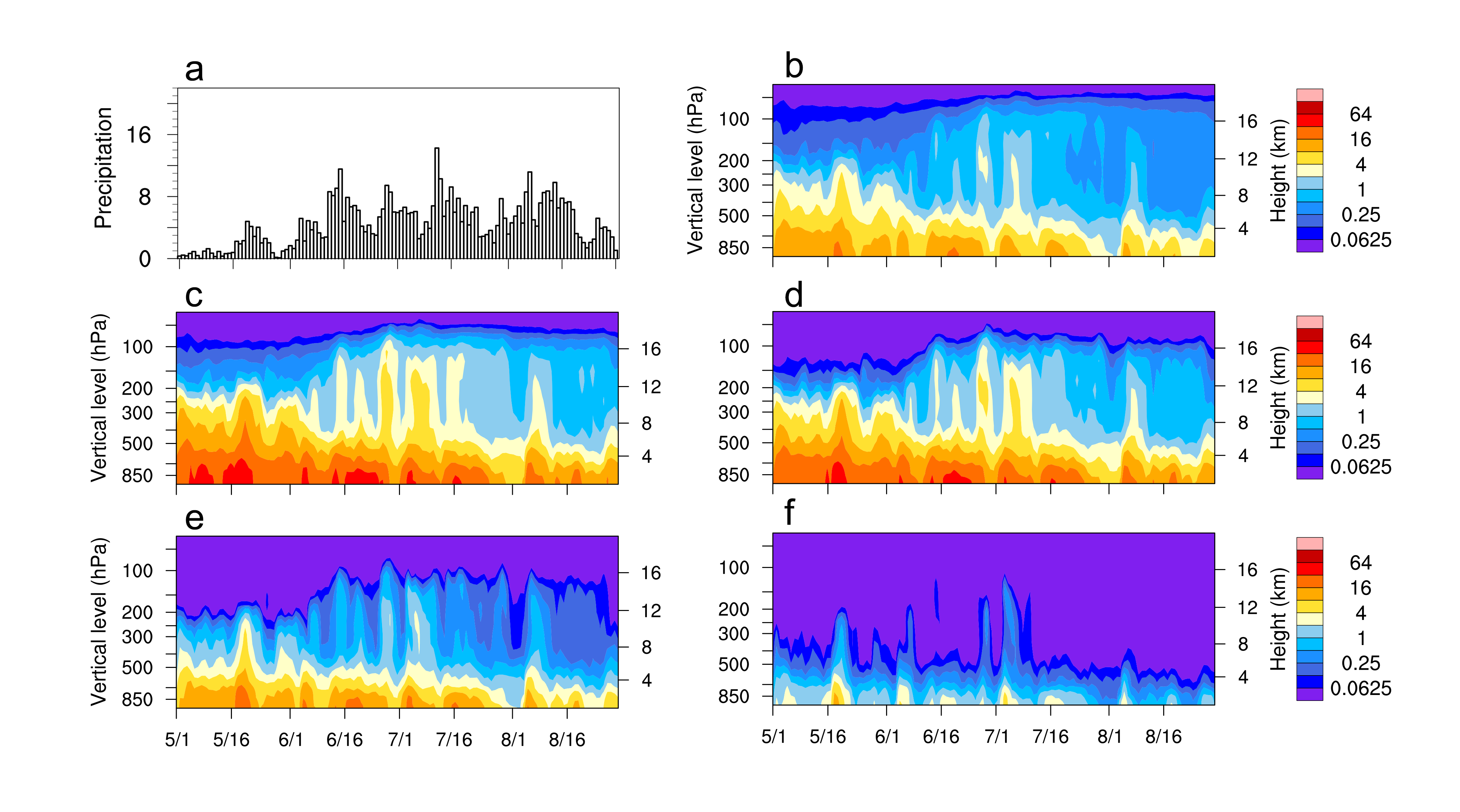


Figure S4 Daily time-height cross- section of of a) precipitation, b) very fine dusts (0.1-1 μ), c) fine dusts (1-1.8 μm), d) fine dusts (1.8 -3μ m), e) coarse dusts (3-6 μm) and d) very coarse dust (6-10 μm) for the Himalayas-Gangetic Plain. Units of precipitation in mm day ^-1^ and dusts in ppbm. Plots are generated using the NCAR Command Language (Version 6.4.0, [https://www.ncl.ucar.edu](https://www.ncl.ucar.edu/), 2017).


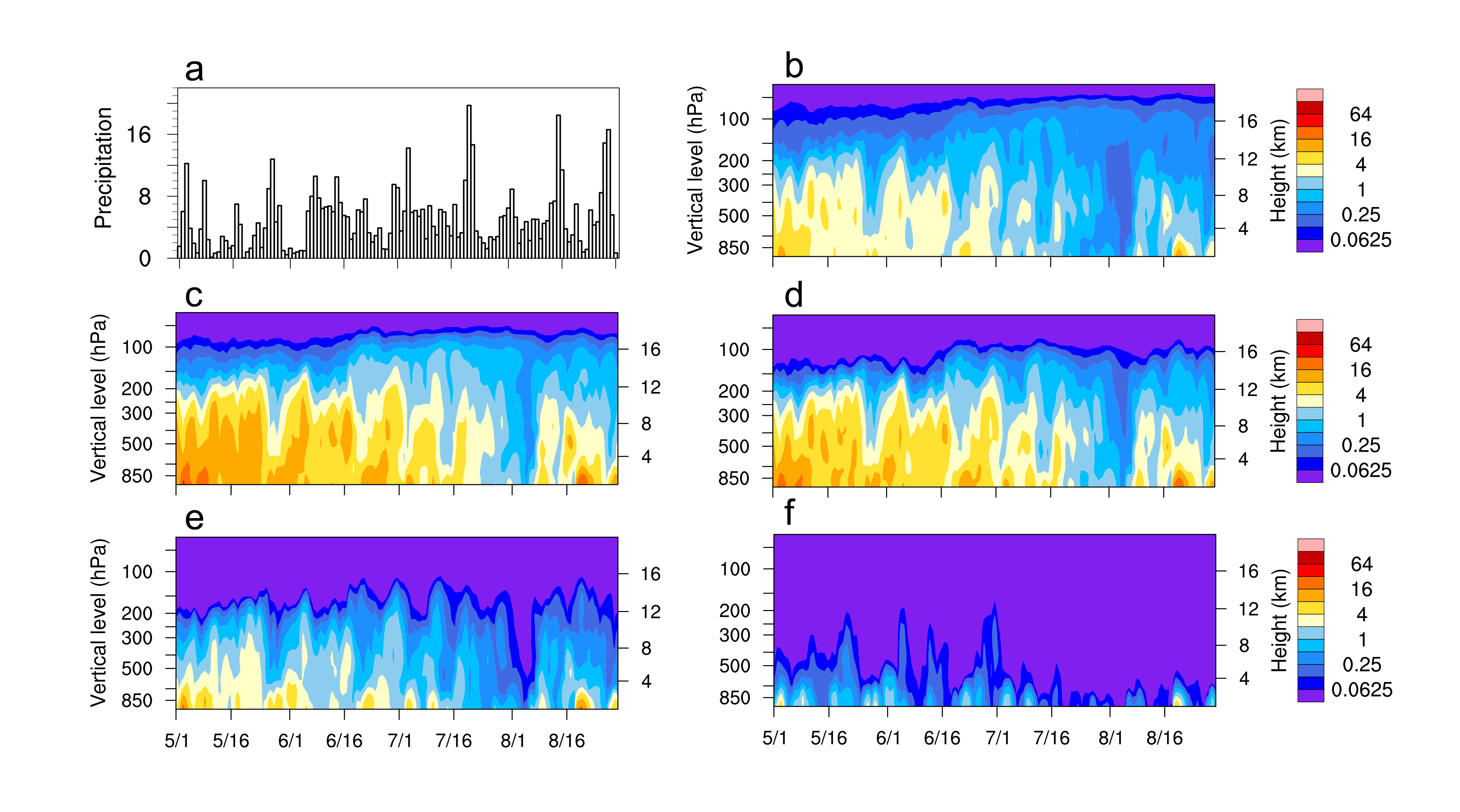


Figures S5 Same as in Fig. S4, except for the Sizhuan Basin
